# Aphthous Sore Mouth in Adults

**Published:** 1835

**Authors:** 


### III. Aphthous Sore Mouth in Adults.

The following cases, contained in a letter from Dr. E. Gaston, of Morristown, Ohio, to Dr. J. C. Bennett, have been transmitted to us, for publication. We hope they will not be found withot interest by the younger members of the professon.

"Agreeably to your request, I send you an account of some of the cases of aphthous sore mouth, occurring in adults, that have fallen under my care.

The first case that came under my notice occurred in

March, 1827. The subject of it was a female, of, I suppose, about twenty-five years of age. At the occurrence of the disease she had been confined in the puerperal state, about two weeks; during which time she had been under the care of Doctor A. When her mouth got sore, she became dissatisfied with him, alledging as the cause of her dissatisfaction, that he had given her mercury without her knowledge, and had salivated her. The doctor protested that he had not given her a particle of mercury in any shape, and professed entire ignorance of the cause of her mouth becoming sore.

He prescribed for her mouth, assiduously, for some days; but finding that all his local and constitutional means did not arrest the disease, and his patient becoming daily more dissatisfied, he, in order to satisfy her, and to remove the imputations with which she branded him, proposed to have me called in to see her, which was accordingly done.

On visiting her, I found her in a truly distressing situation. Her mouth was covered on the inside with aphthæ; lips and tongue considerably swollen; stomach irritable, with frequent fits of vomiting; bowels irregular, sometimes constipated, at others profuse diarrhœa; countenance haggard, and expressive of extreme anxiety; emaciation great; debility such that she could not turn in bed without assistance; and in addition to all her other sufferings, she was, an hour or so before I first saw her, attacked with a profuse hæmorrhage at the nose.—Such was the state of our patient at the time I first saw her; and she attributed all her sufferings at that time, to the state of her mouth.

*Treatment.*—We applied a blister to the back of her neck, in order to arrest the hæmorrhage, and prescribed a dose of calcined magnesia with rhubarb, to clear the bowels of their acrimonious contents. After the cathartic had operated, we prescribed the following course of tonic, alterative and local treatment. *Tonic:* Take cinchona and elixir vitriol, each ℥i, water 1 pint; mix; dose, ℥i four times a day. *Alterative:* Take 6 gr. pill hydrarg. every other night; to be worked off next morning, by a portion of calcined magnesia and rhubarb.

*Local:* Take honey any quantity, and add to it as much muriatic acid as will make pleasantly sour; take a large teaspoonfull, and let it dissolve in the mouth, every three hours; and rinse the mouth out well with a strong decoction of cinchona three times a day. Diet light and nutritive.

On examining her mouth three or four days after my first visit, I discovered that a large slough had come out of her left cheek; but the appearance of her mouth otherwise was much improved. The above treatment was continued, during her recovery—which was rapid and permanent. In two weeks her mouth was well, and her strength so far regained that she could set up an hour or so at a time.

Case 2nd. Occured May, 1829. The subject of this case was a female aged 30 years. She informed me, that for some time previous to her mouth becoming sore, she had been very much harrassed with dyspeptic symptoms, such as loss of appetite—pain and swelling of the stomach after eating—sour and fœtid eructations—irregular bowels, &c.; which symptoms she attributed to travelling, change of water, &c., (as she had but lately emigrated from Louden co. Va.) At this time, her lips, cheeks, and edges of the tongue were covered with white vesicles—centre of the tongue of a dark cherry red color—discharge of saliva profuse and very fœtid. She said that her mouth had been sore for four or five weeks—and if she had taken any medicine, she would have been sure that she had been salivated; but as it was, she was satisfied it could not be the effect of mercury. She had tried every thing that she could hear of, in order to cure it, but all her remedies had proved unavailing. After the aphthæ remained a few days, they would fall off, and then for a short time her mouth would appear to improve, but a fresh crop would arise, and then matters would be as bad as ever.

*Treatment.* Take 6 gr. of pil. hydrargyri every night, and as much calcined magnesia with rhubarb every morning as would open the bowels gently, if the blue pill failed in doing so. As a gargle I prescribed the following: Take sul. zinci ʒi. water half pint, mix. Wash the mouth out with it frequently through the day.

In about a week I had the satisfaction of seeing her comparatively free from all complaints; and a perseverance in the same course for a week longer, effected a cure.

Case 3d. February 27th, 1830. I was requested to visit Mr. E. B. On arriving at his house, he informed me that on the 25th he had gone to a circular fox-hunt; the day was very cold and raw, and the duties he had to perform, required him sometimes to walk till he was very warm, and at others to stand till he would get very cold; in consequence of which he caught cold, and was attacked with symptoms of pleuritis that night. But as he had had similar attacks previously, and had relieved himself by using his own domestic remedies, he thought it unnecessary to apply to a physician for it. But on the night of the 26th he discovered that his mouth was swelling and felt sore—which continued to increase till morning—at which time the saliva literally ran from his mouth, and was so fœtid that he could scarcely endure the smell of it himself. His mouth becoming so sore, he said, had alarmed him, and induced him to send for me. He attributed it to a dose of calomel that he had taken ten years previous to that time, which he thought had lain dormant in his system till he caught cold, and was now venting its fury in salivating him. His lips, cheeks and tongue were considerably swollen, and covered with aphthous spots—flow of salvia still profuse and fœtid—pleuritic symptoms still bad.

*Treatment.* For his pleuritic symptoms, I prescribed the common remedies. For his sore mouth, a watery solution of sulphate of zinc, which relieved him in a few days.

These cases are given as a sample of the cases of the disease that I have seen and prescribed for. I could have given you the history of a number more, but I think it unnecessary, as the symptoms and treatment in the others correspond with these. In these I have advanced no opinion as to the pathology; all I aimed at doing, was to give a correct history of the symptoms and treatment, and let every one draw his own inferences as to the pathology."

